# Some Experiences in the Spread of Scarlet Fever

**Published:** 1906-11-17

**Authors:** Alfred Greenwood

**Affiliations:** Medical Officer of Health for Blackburn County Borough, Lecturer on School Hygiene, Blackburn Technical Schools, Medical Superintendent Blackburn Fever and Small-pox Hospitals


### Hospital Clinics.

### SOME EXPERIENCES IN THE SPREAD OF SCARLET FEVER.

By ALFRED GREENWOOD, M.D., D.P.H., Medical Officer of Health for Blackburn County Borough, Lecturer on School Hygiene, Blackburn Technical Schools, Medical Superintendent Blackburn Fever and Small-pox Hospitals.

Probably in that special branch of medicine known as Preventive or State Medicine, the subject of infectious diseases is more closely associated with the work of the general medical practitioner and that of the medical officer of health, than any other.

Of the notifiable infectious diseases perhaps scarlet fever gives as much trouble to the medical man as any, and several extensive epidemics of this disease have occurred in England during recent years. For these reasons the writer considered that some personal views and experiences of scarlet fever might be of interest, care being taken to avoid anything of a dogmatic nature.

The relationship of the general practitioner to scarlet fever differs now from that which obtained more than fifteen years ago. Formerly isolation hospitals were scarce, and the majority of cases were treated at home. Now, in many instances, all the medical attendant gets is the notification fee of half-a-crown instead of, perhaps, five or ten guineas for medicine and attendance as compared with former days. And here I would pay a tribute to the loyalty and co-operation exhibited by medical practitioners in "taking the bread out of their own mouths" in order to further the aims of preventive medicine.

During the last four or five years considerable doubt has been thrown by certain medical men upon the efficacy of fever hospitals in checking the spread of scarlet fever. So far as I know, no doubt has been cast upon their success in checking typhoid fever and diphtheria. It was natural that these expressions of doubt should cause much questioning on the part of the ratepayer as to whether fever hospitals for scarlet fever produced results commensurate with their cost. Indeed, in certain districts the erection of such institutions has been delayed, and the hospital isolation of this disease has become a burning question.

Those who believe that fever hospitals have failed in stamping out scarlet fever have employed the statistical method, but, on the other hand, the statistical method has been employed by other eminent men which have produced directly opposite results. In any case, the majority of fever hospitals are less than twenty years old, and this period is not long enough, in my opinion, for the purpose of proving that they have been a failure. There are too many fallacies. I think all will agree that removal to hospital of an early case of scarlet fever has evidently prevented the occurrence of that disease amongst the remainder of the household in many instances, and it is well known that the longer the incidence of scarlet fever in any given child can be postponed, the less is the liability to contract the disease, and the greater the chance of recovery if it is contracted.

It seems, therefore, unwise to compare, by statistics, one town with another regarding the success or otherwise of its particular fever hospital in checking scarlet fever. The conditions are so variable as to vitiate scientific comparison. For example, towns differ in size, type of population, and housing conditions. Also the cyclical incidence appears in different towns during different years. Moreover epidemics of the disease itself vary greatly in extent and severity. I have known an epidemic of scarlet fever short in duration, but fatal, and I have also known an epidemic lasting twelve months, with a comparatively small number of fatal cases.

Therefore, in arriving at a decision regarding this important matter, I believe firmly that each town should be considered on its own merits, and for this purpose statistics are of use.

Again, it has been stated that any possible benefit which hospitals may produce is counterbalanced by the occurrence of "return cases" which is a term employed to indicate re-appearance of scarlet fever infection in a household within one month after the return home of a recent inmate of the hospital. It is well-known, however, that return cases occur amongst home-treated cases. This will be shown in the case of Blackburn.

It is, of course, unfortunate that our knowledge of the bacteriology of scarlet fever is so limited, and the whole profession would hail gladly some means of deciding definitely that any case of scarlet fever was free from infection or not, such as we possess in the bacteriological examination of nose and throat swabs from diphtheria patients. At the present time the trouble is that we can never be absolutely sure that any convalescent case of scarlet fever is free from infection. Thus, as we are working in the dark, to some extent, we can only act with all the discretion and care possible in declaring cases reasonably free from infection.

In these days of serum-therapy, perhaps, when the actual cause of scarlet fever has been established, we may have a discovery of some successful curative serum as in diphtheria. Incidentally, I may say that my own experience with the polyvalent serum in twenty septic cases of scarlet fever has not been at all satisfactory. It is true that the number of cases is small, but the results did not encourage me to adopt it as a routine measure. It is possible that the polyvalent serum might be more satisfactory in cases of scarlet fever which contained an excess of those particular cocci which the serum was designed to counteract. It is a fact that I have seen the use of antidiphtheritic serum followed by good results in a few cases of scarlet fever with bad septic throats, in the swabs from which no diphtheria bacilli could be found.

Statements have also been made to the effect that return cases are due to "hospitalisation" of the infecting case. That this is not so appears to be proved by the observations of Dr. Chapin, who isolated a large number of scarlet fever at their own homes, and quarantined the non-infected children in other houses. After the recovery of the patients the healthy children were sent home, yet a considerable number of secondary cases arose amongst the latter, showing that prolonged infectivity and not hospital influence was the cause.

A suggested cause of these return cases is that the breath of the first case, which has been kept in a ward, or, if nursed at home, in one room for a long time, is infectious. Whether this is so or not, a patient who has been declared free from the disease should not be allowed to sleep with other children who are well.

Possible infectivity of the urine of the infecting case has been regarded as another cause.

Also inefficient disinfection of the patient's clothes or hair has been another suggested explanation, but in these days of thoroughness in this respect I do not think that this can be regarded seriously.

Whatever be the cause of return cases of scarlet fever, and this must remain obscure until a fuller bacteriological knowledge of the disease is obtained, I do not think they can be avoided altogether, whether cases are taken to hospital or nursed at home. One can only be as strict and careful as possible.

That there is a fallacy in always blaming a discharged case from the hospital for causing return cases may be illustrated by the following experiences.

A girl aged seven years was removed to the Blackburn Fever Hospital with scarlet fever on the second day of the disease. Disinfection was carried out at the patient's home the same day. The girl had no complications, and remained in hospital for forty-three days, and was then sent home, all due precautions being carried out before discharge. This was on a Friday. On the following Tuesday the only other child at home, a girl aged five years, was taken to the fever hospital with an extremely severe attack of scarlet fever. Naturally the parents thought that the elder girl was the cause of her sister's illness, although they had not occupied the same room, but after a very close inquiry I found that on the Sunday after the first case came from hospital, she had worn her best frock which had previously been exposed to infection, and which had not been given up in order that it might be disinfected by steam, for fear that the process would damage it.

Again, on eight occasions I have marked a patient as fit for discharge from hospital for the day following, when from some sudden cause, such as a rise of temperature, etc., the patient was not allowed to go home, but was kept for a further period of three or four days for observation. In each of these eight families another case of scarlet fever occurred at home within those three or four days, and had the first cases gone out they certainly would have been alleged to be the cause of the second case. Therefore a certain proportion of alleged return cases are due to coincidence.

It is an unfortunate fact that the mortality amongst return cases of scarlet fever is higher than the average. Also the infectivity rate appears to be greater in cases which have been kept in hospital for two months and upwards, than amongst those detained for a shorter interval.

It is probable that in the past there have been certain defects in the administration of fever hospitals. For example, during an epidemic any overcrowding of patients must produce a concentration of the infection, especially if acute and convalescent patients are mixed together. Therefore all overcrowding should be avoided, and convalescent patients should be kept apart so that there will be no opportunity for any reinfection by acute cases. This principle should be followed in the erection of new fever hospitals, and the tendency should be to provide a larger number of wards for a smaller number of patients than a smaller number of wards accommodating more patients in each.

Before cases are discharged—and this applies to those nursed at home as well—particular attention should be paid to bathing, to scrubbing the head, and to the mucous membranes, especially the nose, even if no discharge can be seen.

It should be remembered that a nurse may carry infection from one child to another in the same wards, and at the Blackburn Fever Hospital the nurse is made to wash her hands in disinfectants between the attendance upon each patient, and separate spatulas, etc., are used for each patient, and are disinfected daily. Care is taken that infection shall not be carried by cuffs, etc. Therefore I would say that in my experience, although a fever hospital may not eradicate scarlet fever, it can, and does, if properly managed, check its spread and save life. Few things are perfect in their results.

In a large cotton manufacturing town the isolation and nursing of scarlet fever patients at home is more often unsatisfactory than not. Removal to the hospital of these cases frequently ensures (1) removal of foci of infection; (2) protection of the remainder of the household; (3) better feeding and nursing of the patients. And one should remember that indirect beneficial results also ensue, such as avoidance of wear and tear on the part of the nursing mother for several weeks. I think it is absolutely necessary to remove to hospital all cases of scarlet fever occurring in connection with milk, food, and clothing business.

Although I believe that fever hospitals are an indispensable portion of the community, I do not think it wise to provide hospital accommodation for every case of scarlet fever which occurs. There are undoubtedly some cases which can be isolated satisfactorily at home, such as those occurring in large households where the services of trained nurses are available. Also, in certain smaller households where the patient is the only child and where an intelligent mother can spend her time in attendance, I do not advocate removal to hospital.

Again, removal to hospital often means that the patient can obtain more fresh air when convalescent than he can at home, and here I would say that in my experience it has never been necessary to invoke the aid of the law in enforcing hospital isolation for a case of scarlet fever which could only remain at home to the danger of the public. Indeed, I have only had to threaten it once, and this was at a farm. Even if the parents had continued to hold out I would have stopped the milk supply rather than have adopted the other extreme measure.

For the last three years I have analysed and compared the return cases amongst hospital patients at Blackburn with those occurring at homes from which the patient was not removed to hospital. I have also compared the death-rates amongst hospital and non-hospital patients.

The relative frequency of secondary cases in houses from which the patient was (or was not) removed to hospital during the years 1903, 1904, and 1905, was as follows:—Out of 1,703 cases removed to hospital there were 71 return cases, or a percentage of 4.1. Out of the 683 cases not removed there were 57 return cases, or a percentage of 8.3. The case mortality amongst patients removed to hospital compared with those treated at home during the years 1903, 1904, and 1905 was as follows:—Out of the 1,703 cases removed to hospital there were 69 deaths, giving a case mortality of 4 per cent. Several of these cases which died were in a hopeless condition on admission. Out of the 683 cases not removed there were 33 deaths, giving a case mortality of 4.8 per cent.

These figures would seem to favour removal of scarlet fever patients in Blackburn to the hospital.

The following statement shows the number of scarlet fever cases in Blackburn during the several epidemic years, and also the deaths, death-rates, and case mortality per cent.:—

| Year. | No. of cases. | No. of deaths. | Death rate per 1,000 of the population. | Case mortality per cent. |
|---|---|---|---|---|
| 1887 | 1,695 | 157 | 1.38 | 9.2 |
| 1900 | 1,476 | 83 | .65 | 5.6 |
| 1901 | 1,117 | 58 | .45 | 5.1 |
| 1905 | 1,578 | 76 | .57 | 4.8 |

These figures show that since the opening of the hospital in 1894 the case mortality has been decreasing steadily. But it is possible that the type of scarlet fever, speaking generally, is milder than it was several years ago, and the above improvement in the death-rate may be partly due to this cause; but in spite of this it is possible for a very severe form to occur again.

During the recent epidemic in Blackburn I came across several cases of scarlet fever in which there was no rash, or only a rash of extremely short duration. These gave rise to other cases. One case—that of a nurse—who commenced with nausea, headache, slight pyrexia, and sore throat, was examined four-hourly for the rash, but none was seen. Nevertheless she desquamated as if there had been a well-marked rash of scarlet fever. During the last two years the secondary cases of scarlet fever have been investigated in greater detail, and have been arranged in three groups as follows:—

*Group I.—Cases of Scarlet Fever occurring in a house within one month after the discharge from Hospital of a previous case.*

There were 58 such cases during 1904 and 1905.

The periods respectively between the first case returning home and the infected case were as follows:—

| Period. | No. of Cases. | Period. | No. of Cases. |
|---|---|---|---|
| 1 day | 1 | 13 days | 2 |
| 2 days | 3 | 14 ,, | 1 |
| 3 ,, | 3 | 15 ,, | 1 |
| 4 ,, | 5 | 16 ,, | 3 |
| 5 ,, | 6 | 17 ,, | 1 |
| 6 ,, | 6 | 18 ,, | 1 |
| 7 ,, | 10 | 20 ,, | 1 |
| 8 ,, | 3 | 22 ,, | 1 |
| 9 ,, | 1 | 23 ,, | 1 |
| 10 ,, | 3 | 24 ,, | 1 |
| 11 ,, | 2 | 25 ,, | 1 |
| 12 ,, | 1 | | |

The average number of days between the first case returning home or the infecting case and the second case or infected case occurring was 5.6.

It will therefore be seen that the fourth to the seventh day was the commonest period in this series for an infecting case to cause a return case, and that the greatest number of return cases occurred on the seventh day after the infecting case had arrived home from the hospital.

In investigating the cause of these return cases, a large proportion of the infecting cases showed a discharge from the nose, and in some of these the discharge appeared for the first time after the patient had left the hospital. In this connection it is possible that the custom of bathing a patient immediately before discharge from hospital may tend to the production of catarrh.

*Group II.—Secondary cases occurring in a house from which the first case was removed to Hospital, but which occurred before discharge from Hospital of the first case.*

There were 101 cases during 1904 and 1905. The periods respectively between the onset of the first case and the onset of the second case were as follows:—

| Period after removal to Hospital. | No. of Cases | Period after removal to Hospital. | No. of Cases. |
|---|---|---|---|
| 1 day | 4 | 15 days | 3 |
| 2 days | 5 | 16 ,, | 3 |
| 3 ,, | 13 | 18 ,, | 6 |
| 4 ,, | 14 | 19 ,, | 1 |
| 5 ,, | 3 | 20 ,, | 1 |
| 6 ,, | 8 | 21 ,, | 3 |
| 7 ,, | 5 | 22 ,, | 2 |
| 8 ,, | 5 | 24 ,, | 1 |
| 9 ,, | 4 | 25 ,, | 1 |
| 10 ,, | 4 | 26 ,, | 1 |
| 11 ,, | 2 | 27 ,, | 1 |
| 12 ,, | 2 | 28 ,, | 1 |
| 13 ,, | 7 | 31 ,, | 1 |
| 14 ,, | 1 | | |

The average number of days between the onset of the first case and the onset of the second case was

5.2. The greatest number of cases occurred on the third and fourth days after the first case had gone to hospital, and were probably due to infection received from that first case before isolation.

*Group III.—Secondary cases occurring in a house in which the first case was nursed at home.*

There were forty-four of such cases during 1904 and 1905. The periods respectively between the onset of the first case and the onset of the second case were as follows:—

| Period. | No. of Cases. | Period. | No. of Cases. |
|---|---|---|---|
| 1 day | 2 | 19 days | 1 |
| 2 days | 3 | 20 ,, | 2 |
| 4 ,, | 2 | 21 ,, | 2 |
| 5 ,, | 1 | 26 ,, | 1 |
| 6 ,, | 2 | 28 ,, | 1 |
| 7 ,, | 5 | 29 ,, | 1 |
| 10 ,, | 3 | 30 ,, | 1 |
| 11 ,, | 2 | 37 ,, | 1 |
| 14 ,, | 1 | 41 ,, | 2 |
| 15 ,, | 1 | 42 ,, | 1 |
| 16 ,, | 2 | 51 ,, | 1 |
| 17 ,, | 2 | 58 ,, | 1 |
| 18 ,, | 2 | 65 ,, | 1 |

The average number of days between the onset of the first case and the onset of the second case was 14.8.

Regarding the spread of scarlet fever, it seems probable that desquamating epithelium is not the cause, at any rate not in the same way as the nasal discharges. I remember hearing of one instance in which a child at school desquamating from scarlet fever passed round the class large flakes of skin from her hand, which the other children tasted like a new form of toffee. Yet no subsequent cases occurred in that class. At the same time I believe that the presence of desquamation should be regarded as indicating the presence of infection in the system, perhaps existing in another situation, and perhaps not.

In certain epidemics the desquamation has been so free among some children attending school that it must have come into close contact with other susceptible children, and also have impregnated the dust of the schoolroom to such an extent that large outbreaks of the disease would have been caused if the skin which peels off is always infectious. It is more likely that discharges from the nose, throat, and ear are the most potent causes of the spread of scarlet fever. I think it is very probable that there are "carrier cases" of scarlet fever just as there are of diphtheria—that is, people, although well themselves, who carry the infection—and that this factor may prolong an outbreak considerably. It is to be hoped that the abolition of slates and slate pencils from schools will tend to diminish not only diphtheria but scarlet fever.

The following is an interesting example of the spread of scarlet fever from infected milk. In 1900, when medical officer of health for Crewe, a large number of cases of scarlet fever occurred within a few days which had a common milk supply. Visits to the house of the milk-retailer and the homes of his employers failed to reveal any source of the mischief. Accordingly I visited the farm, which was a large one, situated five miles from the borough, and examined the men and boys employed there. They showed no clinical signs of infection, but a history of sore throat a month before was obtained from a youth aged seventeen years. This was not associated with any other symptoms of illness at his home, according to his statements. I then visited his home about one mile from the farm, and found four children in various stages of scarlet fever, having previously obtained the sanction of the medical officer of health for that district to investigate the outbreak. The youth aged seventeen was prevented from milking, and the outbreak in Crewe ceased immediately.

The above incident shows how necessary it is that all those engaged in milking should report at once any illness at their own homes.

Another valuable aid towards the prevention of scarlet fever is the early notification of cases of this disease. But an obstacle in this connection is the unfortunate habit which many parents have of looking at a rash for a few days, and then asking the medical attendant to see the case when the rash has disappeared. Under such circumstances he has frequently to wait until some other signs, such as desquamation, appear. But even worse than this, many parents never call in a medical man at all. During the last epidemic in Blackburn many cases occurred which showed culpable negligence on the part of parents, in this respect, in an extreme degree. I feel sure that this has been the experience in other towns. There is no doubt that public elementary schools contribute to the spread of scarlet fever, and of course this is in part due to the fact that these institutions contain a very susceptible portion of the population in any community. Constant school inspection, with the co-operation of the teachers, will tend to prevent this, and school closure for this disease ought not to be necessary with ordinary precautions. In this respect, of course, measles is entirely different, the short incubation period, together with the early appearance of the rash, rendering scarlet fever more easy to deal with.

I have worked out the percentages of scarlet fever occurring amongst school children in Blackburn for a number of years. They are as follows:—

*Per cent. of the Total Number of Notifications.*

| | | | |
|---|---|---|---|
| 1897 | 67.5 | 1902 | 60.5 |
| 1898 | 67.7 | 1903 | 49.2 |
| 1899 | 61.3 | 1904 | 65.9 |
| 1900 | 66.0 | 1905 | 66.7 |
| 1901 | 69.8 | | |

It is important that school teachers should have some knowledge of the symptoms of infectious diseases, for, after all, they are the first line of defence in preventive measures at school. Any steps which will further educate the parents as to their duties and responsibilities in this respect are also desirable. Children should be taught to breathe through the nose, and any obstructions to this should be remedied.

In conclusion, I would say that it is by a combination of different measures through which we may hope to diminish the incidence and mortality of scarlet fever, rather than by trusting to one measure only, however important that one may be.

